# Assessing the impact of recombination on the estimation of isolation-with-migration models using genomic data: a simulation study

**DOI:** 10.5808/gi.23016

**Published:** 2023-06-30

**Authors:** Yujin Chung

**Affiliations:** Department of Applied Statistics, Kyonggi University, Suwon 16227, Korea

**Keywords:** coalescent, gene tree, isolation-with-migration models, recombination, simulation

## Abstract

Recombination events complicate the evolutionary history of populations and species and have a significant impact on the inference of isolation-with-migration (IM) models. However, several existing methods have been developed, assuming no recombination within a locus and free recombination between loci. In this study, we investigated the effect of recombination on the estimation of IM models using genomic data. We conducted a simulation study to evaluate the consistency of the parameter estimators with up to 1,000 loci and analyze true gene trees to examine the sources of errors in estimating the IM model parameters. The results showed that the presence of recombination led to biased estimates of the IM model parameters, with population sizes being more overestimated and migration rates being more underestimated as the number of loci increased. The magnitude of the biases tended to increase with the recombination rates when using 100 or more loci. On the other hand, the estimation of splitting times remained consistent as the number of loci increased. In the absence of recombination, the estimators of the IM model parameters remained consistent.

## Introduction

The estimation of divergence between populations and species is a central topic in population genetics and evolution. The difficulty in determining divergence from genetic data is due to the presence of opposing evolutionary processes. For instance, genetic drift increases divergence, and gene flow reduces it [1,2]. The isolation-with-migration (IM) model is a widely adopted demographic model that seeks to reconcile conflicting signals. It models the divergence of two populations from a common ancestral population at a specific time in the past, and accounts for the exchange of migrants between the two populations [1-4].

DNA sequence alignments are commonly used data in the study of IM models [2,5]. IM models typically assume that alignments are shaped exclusively by neutral evolutionary processes and do not take into account the influence of selection, with an absence of recombination within a locus and free recombination between loci [5]. It is deemed essential to confirm that the DNA sequence alignments are orthologs, because the relationship between homologous DNA sequences is believed to be result from past branching processes [6]. To minimize the potential for recombination within a locus, filtering using a four-gamete test [7] should be employed. With the increasing availability of data from the nuclear portions of genomes, it has become possible to obtain data with a history that may include recombination events [6]. However, the four-gamete test has limitations; it may not detect all recombination events [6,7], and methods for filtering genomic data based on four-gamete test results have not been thoroughly evaluated.

Hey and Wang [6] conducted a simulation study on the effect of four-gamete filtering on IM model inference using the IMa3 program [8]. To assess the impact of the four-gamete test, they compared three methods of non-recombined block sampling: the longest interval, overlapping interval, and random non-overlapping interval sampling. The results showed that the distributions of the migration rate parameters were flatter with recombination, and the distribution of the maximum *a posteriori* (MAP) values shifted to the right. This implies that using four-gamete filtering reduces the statistical power for detecting non-zero migration rates. They also found that random intervals performed better, although using the longest interval led to higher-resolution results because of using more data. These results can be applied to genealogy-sampling-based methods for IM models or subsets of IM models.

The study conducted by Hey and Wang [6] provides a significant opportunity for further investigation into the impact of recombination and the filtering method. First, it is important to examine the sources of estimation errors present in their findings. These errors arise from estimating two levels of uncertainty: the distribution of DNA sequences given a genealogy and that of genealogy given an IM model [2,5]. Additionally, errors in detecting recombination breakpoints can also contribute to the overall errors. Hence, it is necessary to understand the source of errors in Hey and Wang’s [6] study and to differentiate between the estimation errors arising from the two levels of uncertainty in the IM model inference and from recombination detection. This information is crucial for improving the accuracy of further studies and fully understanding the impact of the recombination and filtering methods.

Secondly, it is important to extend Hey and Wang’s study [6] to encompass genomic data. As the availability of genomic data increases, software such as MIST [1] has been developed to analyze large amounts of genomic data [2]. However, the study conducted by Hey and Wang [6] focuses on data containing 10–50 loci. Hence, it is imperative to extend the investigation to include an analysis of a larger number of loci and to examine whether the same results obtained by Hey and Wang [6] are observed with a large number of loci. Therefore, it is necessary to evaluate the consistency of the parameter estimators of IM models with a large number of loci.

This study aims to extend the study of Hey and Wang [6] and conduct a simulation-based investigation that examines the impact of recombination on the estimation of IM models using the MIST program. First, this study aims to assess the consistency of the parameter estimators, by analyzing up to 1,000 loci. Second, in order to separate the sources of the errors, we focused on errors arising from the level of uncertainty in the distribution of genealogies given an IM model. To accomplish this, we assumed that recombination breakpoints were known and analyzed the true gene trees of loci determined by the random interval sampling. This study disregarded estimation errors of gene trees and recombination breakpoint detection, focusing solely on the effect of recombination.

## Methods

### The impact of recombination on gene trees

**Fig. 1.**
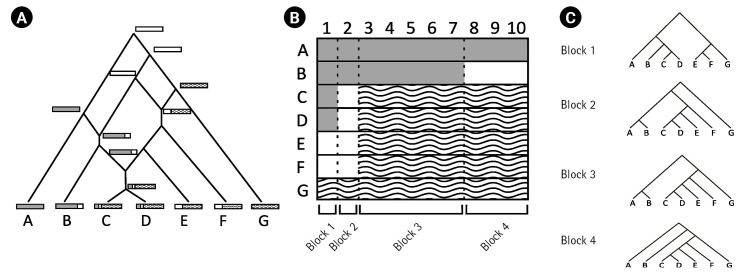
(A) Illustration of a complex evolutionary history with three recombination events. (B) The DNA alignment resulting from the evolutionary history in (A). The alignment is composed of four non-recombined blocks because of three recombination breakpoints. (C) Different gene trees of each of the four blocks in (B).

This study examined how recombination affects the inference of an IM model. Genetic drift, one of the fundamental mechanisms of evolution, results in changes in the allele frequencies in a population by random chance [5]. The evolutionary paths of gene copies by genetic drift can be represented as a gene tree, and the coalescent theory [9] is commonly employed to describe the distribution of the gene tree of a locus. However, the presence of recombination events can complicate evolutionary history (Fig. 1A) and, therefore, affect the inference of an IM model. To isolate the impact of recombination on the IM model inference via gene trees, our study focused on analyzing the true gene trees of loci, which were determined using the true recombination breakpoints.

Recombination events can result in the formation of complex network-like structures. For example, Fig. 1A illustrates the complex evolutionary history of DNA sequences A–G with three recombination events. The first recombination occurred between the white and dashed DNA sequences. The second recombination occurred between the grey and white DNA sequences. Finally, the third recombination occurred between descendants of previsouly recombined sequences. The three recombination breakpoints in alignment A–G (Fig. 1B) produced four non-recombined blocks, each with a different gene tree as a result of recombination events.

In this study, we simulated complex evolutionary histories with and without recombination. Given the true recombination events, we were able to determine the non-recombined genetic blocks and their corresponding true gene trees. We applied random interval samplings based on the results of the study by Hey and Wang [6]. For instance, using the random interval sampling, we analyzed the gene tree corresponding to a randomly selected block from the four blocks defined by the recombination breakpoints in Fig. 1C.

### IM model inference from gene trees

In a 2-population IM model, two populations of effective sizes, *N*_1_ and *N*_2_, have diverged from a common ancestral population of effective size *N*_3_ at generation *t* in the past. The IM model also takes into account migration between the two populations. While *M*_1_ is the proportion of population 1 that is replaced by migrants from population 2 per generation, *M*_2_ is the proportion of population 2 that is replaced by migrants from population 1 in each generation. The IM model provides a comprehensive framework for studying population divergence with migration dynamics between two populations over time. The calculation of the probability of alignments under an IM model is achieved through the integration of two levels of uncertainty: (1) calculating the probability distribution of an alignment given a genealogy using a mutation or substitution model [10-13] and (2) calculating the probability distribution of a genealogy given a demographic model with parameters Ψ using a stochastic process such as coalescent processes [9,14]. Integration over possible genealogies is typically performed using a Markov chain Monte Carlo (MCMC) simulation [1-4,8,15].

In this study, we utilize the MIST program to estimate the demographic parameters of an IM using gene trees. The MIST program implements a two-step analysis to infer the IM model from DNA alignments [1]. In the first step, gene trees of loci without migrations are simulated through an MCMC simulation, without needing any prior information about the demographic model, thereby mitigating the issue of slow mixing. In the second step, the joint posterior density of the demographic parameters in the IM model is estimated from the sampled gene trees, and the MAP estimations of all demographic parameters are obtained. Rather than sampling migrations and the underlying demographic model parameters in the first step, integration over potential migrations is carried out in the second step when the posterior distribution of the IM model is calculated. The MIST program can be used to infer an IM model from true or estimated gene trees [16].

By utilizing the second stage of the MIST program, we calculated the posterior distribution of an IM model from gene trees, with uniform priors for demographic parameters, which served as the likelihood of the IM model in this study. The MAP estimates of the parameters obtained in stage 2 were equivalent to the maximum likelihood estimates of the demographic parameters.

### Simulation setting

In this study, we adopted the simulation setting in Hey and Wang [6]. Similar to Hey and Wang [6], we set the neutral mutation rate per generation per base pair to *μ* = 10^-8^, and hence the mutation rate for the entire locus of length 5,000 base pairs to *u* = 10^-8^ × 5000. We denoted *r* as the probability of a cross-over per generation between the ends of the locus. For clarity and simplicity in notation [15], we use demographic parameters scaled by the mutation rate throughout the remainder of this manuscript (Table 1). Similar to the simulation setting in Hey and Wang [6], the scaled population sizes are assumed to be equal to *θ_i_* = 4*N*_i_*u* = 10 for *i* = 1, 2, 3 in the entire simulation. Splitting time τ = tu changes as 0.5, 2.5, 10 in terms of the number of mutations. The migration rates *m_i_* = *M_i_*/*u* for *i* = 1, 2 are expressed as the number of migrations per mutation. Following Hey and Wang [6], we examine two scenarios: no migration with *m*_1_ = *m*_2_ = 0 and unidirectional migration with *m*_1_ = 0.1 and *m*_2_ = 0 when the splitting time of an IM model is 10 (τ = 10). The recombination rate per mutation *ρ* = *r/u* is varied as 0, 0.2, 1, and 5. It is important to note that *ρ* = 0 indicates no recombination within a locus, which is a typical assumption for the IM model inference.

As this study aimed to assess the consistency of demographic parameters in an IM model, we consider 10, 100, and 1,000 loci for each case. We applied the *ms* program [17] to simulate the gene trees and recombination events. In all cases, gene trees with four tips were simulated as two sequences were sampled from each population at each locus. For each scenario, 100 replicates were generated. When the MIST program was used to analyze the simulated gene trees, we set the uniform priors with upper bounds of 50 for population sizes, 0.2 for migration rates, and 20 for the splitting time for all cases.

## Results

We evaluated the effect of recombination on the distribution of gene trees and IM model inferences. Therefore, we used the true recombination breakpoints and analyzed true gene trees of randomly selected non-recombined loci using the MIST program.

**Fig. 2.**
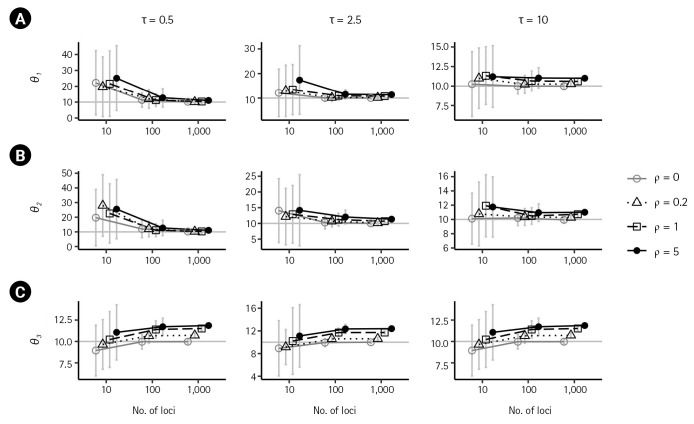
Simulation results illustrating the impact of recombination on population sizes, *θ*_1_ (A), *θ*_2_ (B), and *θ*_3_ (C), as a function of loci. Each plot compares the results in the absence of recombination (*ρ* = 0) with those from low to high recombination rates (*ρ* = 0.2,1,5) when m_1_ = m_2_ = 0 and *θ*_1_ = *θ*_2_ = *θ*_3_ = 10 (represented in a grey horizontal line in each plot). Columns show the results when different splitting times of τ = 0.5,2.5,10 were considered. Bars indicate standard errors, and the x-axis for the numbers of loci is on a log scale. Points with bars were horizontally scattered around the corresponding number of loci to minimize overlap.

**Fig. 3.**
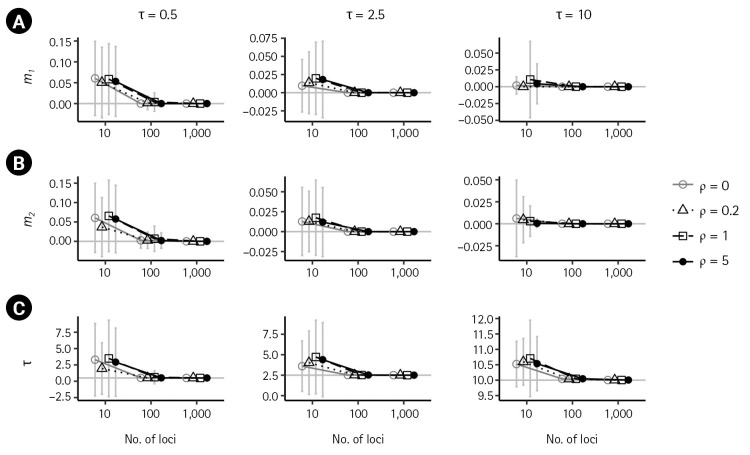
Simulation results illustrating the impact of recombination on migration rates, *m*_1_ (A), *m*_2_ (B), and splitting time (C) when *m*_1_ = *m*_2_ = 0 and *θ*_1_ = *θ*_2_ = *θ*_3_ = 10. Please refer to the Fig. 2 caption for the detailed labels.

**Fig. 4.**
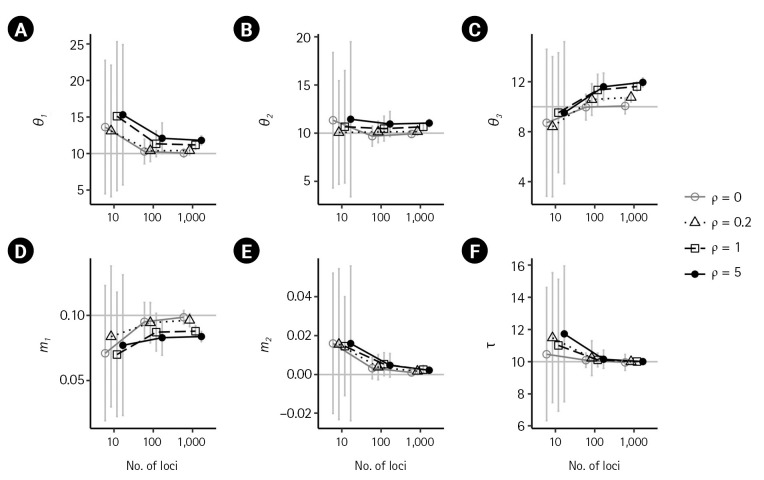
Simulation results illustrating the impact of recombination on isolation-with-migration model parameters. (A–F) The true parameter values of *θ*_1_ = *θ*_2_ = *θ*_3_ = 10, *m*_1_ = 0.1, *m*_2_ = 0 and τ = 10 are indicated by gray horizontal lines. Each plot compares the results in the absence of recombination (*ρ* = 0) with those from low to high recombination rates. (*ρ* = 0.2,1,5). Bars indicate standard errors, and the x-axis for the numbers of loci is on a log scale. Points with bars were horizontally scattered around the corresponding number of loci to minimize overlap.

First, we examined the consistency of the estimators of the IM model parameters in the absence of recombination. (i.e., *ρ* = 0) When there are no migrations (*m*_1_ = 0,*m*_2_ = 0), the estimates of the six parameters converged to their respective true value as the number of loci increased, as shown in Figs. 2 and 3 by grey lines with empty circles. The same consistency was observed when *m*_1_ = 0.1, as depicted by the grey lines with empty circles in Fig. 4. Moreover, in all cases of *ρ* = 0, the standard errors decrease with the number of loci.

In the presence of recombination, the estimates of the IM model parameters were more biased than those in the absence of recombination, regardless of the absence of migration (*m*_1_ = 0,*m*_2_ = 0) or the presence of migration (*m*_1_ = 0.1). For the case of no migrations (*m*_1_ = 0,*m*_2_ = 0), population sizes tended to be overestimated as the recombination rate increased when using 100 loci or more (Fig. 2). In specific, ($\hat{\theta_{1}}$) and ($\hat{\theta_{2}}$) were significantly increased as *ρ* increased (p < 0.001 for the slope of the regression line) at both intermediate and high splitting times, but the increase was only significant (p < 0.001) at the low splitting time (τ = 0.5), as depicted in Fig 2A and B. In all cases of splitting times and the number of loci (Fig. 2C), ($\hat{\theta_{3}}$) was significantly increased with an increasing recombination rate (p < 0.001). In addition, the estimate of the ancestral common population size θ3 (Fig. 2C) exhibited greater bias than estimates of the other two population sizes, θ1 and θ2 (Fig. 2A and 2B). At intermediate splitting time τ = 2.5 and 1,000 loci, the bias of ($\hat{\theta_{3}}$) was 0.6138, 1.7131, and 2.3964 as *ρ* increased from 0.2 to 5. At high splitting time τ = 10, the bias of ($\hat{\theta_{3}}$) was alleviated somewhat as 0.7184, 1.515, and 1.86. The estimates of the migration rates and splitting time appeared to approach the true values with an increasing number of loci, as depicted in Fig. 3. The standard errors of all parameter estimators showed a substantial reduction with an increasing number of loci in the presence of recombination (Figs. 2 and 3).

For the case of *m*_1_ = 0.1, population sizes were significantly increased as *ρ* increased (p < 0.001) when using 100 or more loci (Fig. 4A–C). Moreover, the biases in population size estimations were more severe than those in the case of no migration. For example, at high splitting time τ = 10, the bias of (θ3 ) was 1.59039, 1.6164, and 1.9518. However, the estimate of the non-zero migration rate $\hat{m_{1}}$ was underestimated as the recombination rate increased, despite having a large number of loci (Fig. 4D). The estimated splitting time converged toward the true value (Fig. 4F). The standard errors of all parameter estimators showed a substantial reduction with an increasing number of loci in the presence of recombination (Fig. 4).

## Discussion

This study extends the investigation of Hey and Wang [6]. It conducts a simulation-based investigation to examine the impact of recombination on estimating IM models using the MIST program. In particular, we aimed to assess the consistency of the demographic parameter estimators by analyzing up to 1,000 loci and focus on errors arising from the uncertainty from the distribution of genealogies given an IM model. By assuming that recombination breakpoints are known and analyzing the true gene trees of loci randomly selected from blocks determined by the true recombination breakpoints, we isolated the effect of recombination only.

This study examines the consistency of the estimators of the IM model parameters in the absence and presence of recombination. In the absence of recombination, the estimates of the six parameters converged to their true values as the number of loci increased, and the standard errors decreased with the number of loci. However, in the presence of recombination, the estimates of the IM model parameters were more biased, with population sizes overestimated as the recombination rate increased. The estimate of the ancestral common population size was particularly more biased than the estimates of the other two population sizes. The non-zero migration rate was observed to be underestimated as the recombination rate increased. The splitting time appeared to approach the true value with an increasing number of loci in all cases.

The present investigation yielded findings that are congruent with those of the previous research conducted by Hey and Wang [6], indicating that certain migration rates and population sizes are subject to bias when recombination is present. However, the current study also revealed that the magnitude of such biases tended to increase with an increase in the number of loci. It is worth noting that Hey and Wang [6] reported biases in the estimate of the splitting time using up to 50 loci. In contrast, the current study found that the estimation of the splitting time since divergence was consistent and accurate with a greater number of loci.

Further investigations are required to understand the sources of errors when inferring IM models. In particular, it would be intriguing to assess the accumulated errors that arise from estimating recombination breakpoints from DNA alignments and inferring IM models from DNA alignment analyses, and to compare these results with those obtained in this study. A simulation study could provide insight into whether biases in population sizes and migration rates persist and whether the estimate of the splitting time remains consistent.

This study highlights the importance of considering the effect of recombination in IM model inference and avenues for improving the methodology. The identified biases in population sizes and migration rates serve as valuable information for developing correction methods or adjustment techniques that mitigate the bias introduced by recombination. Moreover, the study underscores the need for future models to explicitly incorporate recombination processes. By explicitly accounting for recombination in the modeling framework, researchers can capture the complexities and nuances of genome evolution more realistically. Furthermore, investigating additional factors, such as the estimation of recombination breakpoints, and integrating them into the model can lead to a more comprehensive and realistic representation of evolutionary processes. By developing correction methods, explicitly incorporating recombination, and exploring additional factors, researchers can construct better genome evolution models that capture the complexities of real-world evolutionary processes.

## Figures and Tables

**Table 1.** Notations and simulation setting

Demographic parameters	Scaled parameters	Description	True values
*N_i_*	*θ_i_=4N_i_u*	Population sizes	*θ_1_=θ_2_=θ_3_=*10
*t*	*τ=tu*	Splitting time	*τ*=0.5, 2.5, 10
*M*	*m=M/u*	Migration rates	*m_1_=*0, 0.1;* m_2_=*0
*r*	*ρ=r/u*	Recombination rates	*ρ*=0, 0.2, 1, 5
